# Determination of reference intervals for symmetric dimethylarginine (SDMA) in Lacaune sheep

**DOI:** 10.1186/s12917-026-06074-7

**Published:** 2026-11-05

**Authors:** Frederik Kiene, Matthias G. Wagener, Petra Röhrig, Martin Ganter

**Affiliations:** 1 Clinic for Swine, Small Ruminants and Forensic Medicine, University of Veterinary Medicine Hannover, Foundation, Hannover, Germany; 2 Field Station for Epidemiology (Bakum), University of Veterinary Medicine Hannover, Foundation, Hannover, Germany

**Keywords:** SDMA, Sheep, Renal biomarker, Creatinine, Kidney disease

## Abstract

Traditional biomarkers such as serum creatinine and urea often fail to reflect early renal dysfunction, particularly in emaciated animals, as they depend on muscle mass or dietary protein intake. Symmetric dimethylarginine (SDMA), a novel renal biomarker, has shown promise in other species for earlier and more sensitive detection of kidney disease. However, reference intervals for SDMA in ovine species remain undocumented. This study aimed to establish reference intervals for serum SDMA in clinically healthy Lacaune sheep. A reference population of 143 healthy sheep, stratified by age (juvenile < 1 year, adult ≥ 1 year), was selected based on clinical health, body condition score, and serum creatinine concentration. Serum SDMA was quantified using an ELISA validated for cats and dogs. Reference intervals were calculated as 0.59–1.72 µmol/l for all sheep, 0.59–1.56 µmol/l for adults, and 0.59–1.83 µmol/l for juveniles, with juveniles exhibiting significantly higher concentrations (*p* < 0.001). No sex-related differences were observed. In a retrospective analysis of 51 deceased sheep with histopathological renal assessments, SDMA concentrations were significantly elevated in animals with severe renal lesions compared to those with no, mild or moderate lesions (*p* = 0.005). Creatinine by contrast did not show significant differences. Notably, in animals with poor body condition, SDMA remained effective in identifying individuals with severe renal lesions, whereas creatinine levels remained within or below the reference interval. These findings suggest that SDMA is a more sensitive indicator of renal dysfunction in sheep than creatinine, particularly in emaciated individuals. The established reference intervals provide a foundation for clinical application. The economic feasibility of SDMA testing remains a limitation, but its potential to improve early diagnosis of renal disease in sheep justifies further investigation.

**Supplementary Information:**

The online version contains supplementary material available at https://doi.org/10.1186/s12917-026-06074-7.

## Introduction

Although generally rare, renal kidney failure in sheep is mainly related to tubular necrosis induced by bacterial, as in the case of pulpy kidney enterotoxaemia (*Clostridium perfringens* type D [38]), chemical and, particularly in the southern hemisphere, plant toxins [1, 19, 41]. Causes for diagnoses of renal insufficiency in routine veterinary practice are, however, almost always attributed to disorders upstream (pre-renal) and downstream (post-renal) of the kidney. Pre-renal kidney failure is caused by a functional hypo-perfusion of the kidneys, which can be based on cardiac insufficiency, major blood loss or dehydration. The latter is often observed in combination with cases of hypocalcaemia, rumen acidosis or ketosis [2, 19, 51]. Post-renal kidney insufficiency is caused by the obstruction of the lower urinary tract resulting in urinary retention with compression of the functional kidney tissue. This can often be observed in cases of obstructive urolithiasis in castrated rams or uterine tumours compressing the urethra of older female sheep [1, 15, 19, 41].

Monitoring renal function is a basic element of the internistic diagnostic process and the management of intensive care patients. Since the correct measurement of the glomerular filtration rate (GFR) is time-consuming and elaborate, concentrations of the plasma components urea and creatinine are often used as approximation parameters for monitoring kidney function [20]. These two traditional parameters have, however, important disadvantages which make them difficult to assess in the context of diagnostics and preclude their meaningful application in many cases [34]. Urea or blood urea nitrogen (BUN), as end product of the amino acid metabolism, is strongly influenced by dietary protein intake and feed composition [4, 22]. Creatinine is a metabolite of the muscular energy metabolism. It´s serum concentration is hence directly proportional to the amount of muscular tissue of an individual [4]. The creatinine plasma concentration can therefore vary within species in relation to breed, age and sex [29, 40]. Beyond that, the close relationship of plasma creatinine to the amount of muscular tissue becomes particularly problematic when the parameter is interpreted in sheep, as these animals, when in intensive care, are often severely emaciated [47]. After the loss of fat reserves, the muscular tissue, rich in protein, is catabolized. This leads to increased blood urea concentrations. At the same time, the small quantity of muscular tissue does not provide a sufficient amount of creatinine to adequately reflect the kidney filtration capacity [35]. Kidney function is only reflected to a limited extent by the creatinine blood concentration. Depending on the species, only when glomerular filtration rate is reduced by more than approximately 75%, plasma creatinine concentration rises above the upper reference limit [6, 24]. Not least due to the inaccuracy of this most commonly used parameter, the incidence of chronic kidney disease is greatly underestimated in both human and veterinary medicine [23].

Along with cystatin-C, symmetric dimethylarginine (SDMA) represents a new generation of renal biomarkers, which are available through the examination of a single blood sample and which promise an early and more sensitive identification of renal dysfunction [20, 24, 44]. In contrast to creatinine, SDMA is a product of all nucleated cells. It is a derivative of the amino acid L-arginine and is formed by methylation within the protein metabolism of the cell nucleus [53]. After proteolysis, SDMA enters the bloodstream and is in humans 90% excreted via the kidneys [43]. Although first described over 50 years ago [26], SDMA has only been interpreted as a kidney parameter in human medicine since the beginning of the twenty-first century [9, 17, 24, 29]. In veterinary medicine, the parameter has been established for the use in cats and dogs around ten years ago [24, 40]. Also studies on serum SDMA in horses [31, 42, 45, 46] and cattle [28] confirm the parameter as a useful tool for the diagnosis of kidney dysfunction in the respective species. In goats, a reference interval (0.4–1.3 µmol/l) was calculated by Camacho et al. [12]. For the application in sheep, no data are available to date.

The present study aims to establish reference intervals for SDMA in sheep in strict accordance with the guidelines of the American Society for Veterinary Clinical Pathology [18]. For this purpose, a reference population of 160 clinically healthy Lacaune sheep was selected. Furthermore, the study has the objective to test the hypothetic diagnostic superiority of SDMA over creatinine in early detection of renal dysfunction in sheep. For this purpose, serum samples obtained from 51 deceased ovine patients with available pathological and histopathological diagnostic data were retrospectively analysed to determine their status of renal function.

## Materials and methods

### Study populations

For the establishment of reference intervals of SDMA in sheep, serum samples were collected from 160 clinically healthy Lacaune sheep (reference population) during summer 2024. The samples were obtained as part of routine Maedi-Visna surveillance and trace element status monitoring in a single dairy herd. The animals studied were kept in a free-range barn with seasonal access to pasture. Samples were selected based on age distribution representative for the farm. For the analysis of demographic influences on SDMA serum concentration and reference interval calculation, individuals of the reference population were further selected based on four predefined health criteria. Selected individuals had to appear clinically inconspicuous upon brief assessment. The body condition, systematically assessed by scoring muscle and fatty tissue dorsal to the lumbar vertebrae (Body Condition Score, BCS; five increments according to [49]), was required to range between 2 and 4. The conjunctivae, systematically scored using the FAMACHA system (five increments; [5]) were to exhibit a score of 2 or lower. Additionally, serum creatinine concentration was required to remain below the upper reference limits published by Bickhardt et al. [8] (lambs: 130 µmol/l) and Tschuor et al. [50] (adults: 104 µmol/l).

For the second part of the study, serum samples from 51 deceased sheep patients presented to the veterinary clinic for small ruminants between 2018 and 2022 (test population) were retrospectively analysed for kidney function parameters (Supplementary File 1). Main selection criterion was the availability of serum that had been obtained less than 48 h prior to the pathological examination. All animals underwent pathological, and histopathological examination by specialized veterinary pathologists. The patient population consists of 18 males and 33 females of various ages (range: 19 days–14 years) and breeds.

### Laboratory analyses

Blood samples were obtained by puncturing the jugular vein (9 ml S-Monovettes, Sarstedt, Nümbrecht, Germany). All samples were centrifuged at 2,000 × g within 6 h of collection. Resulting sera were stored in Eppendorf tubes at − 20 °C. Storage prior to laboratory examination of the reference population did not exceed a period of three months. As the clinical cohort was investigated retrospectively, sera were stored under the mentioned conditions in individual cases for several years (mean: 2.52 ± 1.56 years). All samples (reference population and clinical cohort) were investigated in the in-house laboratory of the Clinic for Swine and Small Ruminants. Serum SDMA concentrations [µmol/l] were determined using a commercially available enzyme-linked immunosorbent assay (ELISA) kit validated for veterinary use (SDMA Vet ELISA, DLD Diagnostika GmbH, Hamburg, Germany). The kit is used for the quantitative determination of derivatized SDMA in canine or feline plasma or serum, in which SDMA is converted into N-acyl-SDMA during sample preparation by an acylation reagent. The assay is based on a competitive enzyme immunoassay. The measured signal at 450 nm after the substrate reaction is hence inversely proportional to the SDMA concentration in the sample. Analyses were performed according to the manufacturer’s instructions. Additional serum biochemical parameters included creatinine [µmol/l], total protein [g/l] and albumin [g/l]. These variables were measured by an analyser (Cobas Mira Plus, Roche Diagnostics, Rotkreuz, Switzerland) according to the clinic’s routine standard procedures, as previously described by Grimm et al. [21] and Wagener et al. [52].

### Statistical analyses

Statistical analyses were performed using the R environment (version 4.4.0; [37]). Results were considered statistically significant at *p* < 0.05.

Differences in SDMA concentrations related to age (juvenile vs. adult) and sex (male vs. female) were assessed using the Wilcoxon rank-sum test. In addition to a data set with all individuals, subsets were subsequently created according to age category. For each dataset (all individuals [*n* = 143], adults only [*n* = 80], juveniles only [*n* = 63]), data distribution was evaluated visually using histograms and Q–Q plots (*ggplot2* and *psych* packages, [39, 54]). Although normality was assessed for descriptive purposes, the primary method for reference interval calculation was nonparametric. Outliers were identified using Tukey’s interquartile fences (*outliers* package, [30]) and excluded where applicable. Limits of the reference interval were calculated as the 5th and 95th percentiles of the non-outlier SDMA values, and central tendency was described using mean and median. To assess the stability of these reference intervals, 90% confidence intervals for the 5th and 95th percentiles were estimated using the bootstrap method (1000 resamples, bias-corrected and accelerated, BCa) implemented in the boot package [13].

For the evaluation of the diagnostic superiority of SDMA over creatinine, histopathological renal findings in the clinical cohort (*n* = 51) were classified into the four categories: no pathological findings (33/51), mild renal lesions (4/51; non-inflammatory, focal alterations), moderate renal lesions (6/51; mild inflammatory or degenerative processes) and severe renal lesions (8/51; generalised, severe pathological findings). Detailed histopathological findings of the cases for each category are listed in Table 1. Demographic data and information on the BCS of the individuals of the clinical cohort is provided in Supplementary Table 1. Differences in concentrations of serum SDMA and serum creatinine between pathological categories were assessed using non-parametric tests due to non-normal data distribution. Pairwise comparisons between groups were performed using the Wilcoxon rank-sum test with continuity correction. *P*-values were adjusted for multiple comparisons using the Benjamini–Hochberg procedure [7]. Measured SDMA values of diseased animals were compared with the here established SDMA reference interval and with an established reference interval for serum creatinine [50]. Additionally, creatinine and SDMA concentrations were graphically assessed in relation to their respective reference intervals across body condition scores. Due to the small number of individuals in the clinical cohort, age-independent reference intervals were used for the assessment of the data.

**Table 1:** Histopathological findings in 18 of 51 individuals from the clinical cohort exhibiting renal lesions deviating from the physiological norm

Renal lesion category	Histopathological findings
mild (*n* = 4) (non-inflammatory, focal alterations)	focal lymphangiectasia
	mild mineralization of tubular lumina
	mild mineralization of tubular lumina
	mild mineralization of tubular lumina
moderate (*n* = 6) (mild inflammatory or degenerative processes)	mild glomerular protein accumulation
	mild focal interstitial nephritis
	mild interstitial nephritis
	moderate tubular mineralization, tubular degeneration and necrosis
	mild interstitial nephritis
	pigmentary nephrosis
severe (*n* = 8) (generalised, severe pathological findings)	autolysis, mineralization of tubular lumina
	tubulonephrosis and interstitial nephritis
	autolysis following *Clostridium perfringens* type D enterotoxaemia
	glomerulonephritis
	autolysis following *C. perfringens* type D enterotoxaemia
	tubular concretions
	renal pelvic dilation, interstitial nephritis, tubular degeneration
	autolysis following *C. perfringens* type D enterotoxaemia

## Results

### Calculation of SDMA reference intervals for sheep

All 160 animals examined showed creatinine concentrations below the upper reference limits of Bickhardt et al. [8] (lambs: 130 µmol/l) and Tschuor et al. [50] (adults: 104 µmol/l). Several individuals showing a BCS below 2 (*n* = 10) and above 4 (*n* = 0), and/or a FAMACHA score above 2 (*n* = 10) needed to be excluded from further investigations, resulting in a valid reference population of 143 animals.

**Fig. 1:**
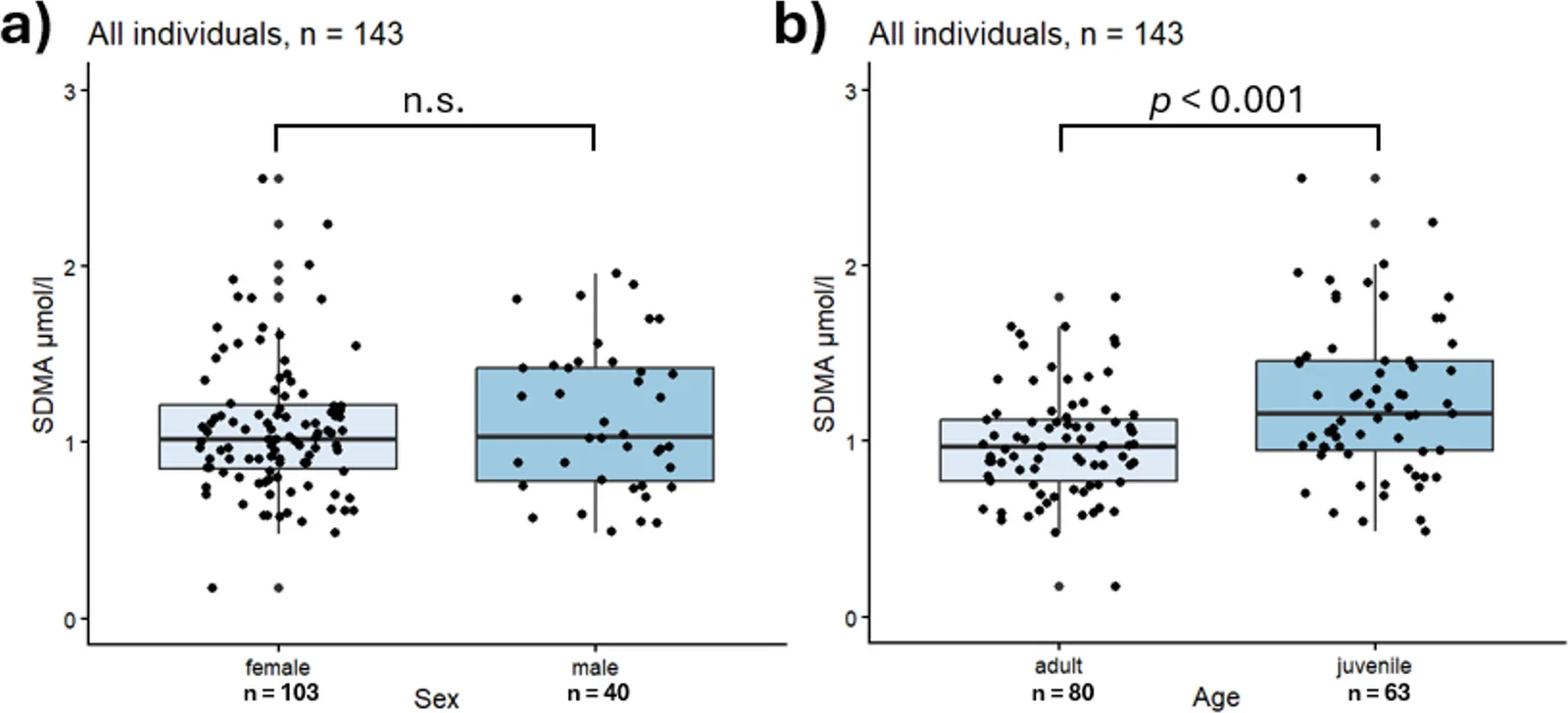
Assessment of the influence of demographic factors on serum symmetric dimethylarginine (SDMA) concentrations in 143 clinically healthy Lacaune sheep. **a** Sex and (**b**) age were evaluated using the Wilcoxon rank-sum test

**Fig. 2:**
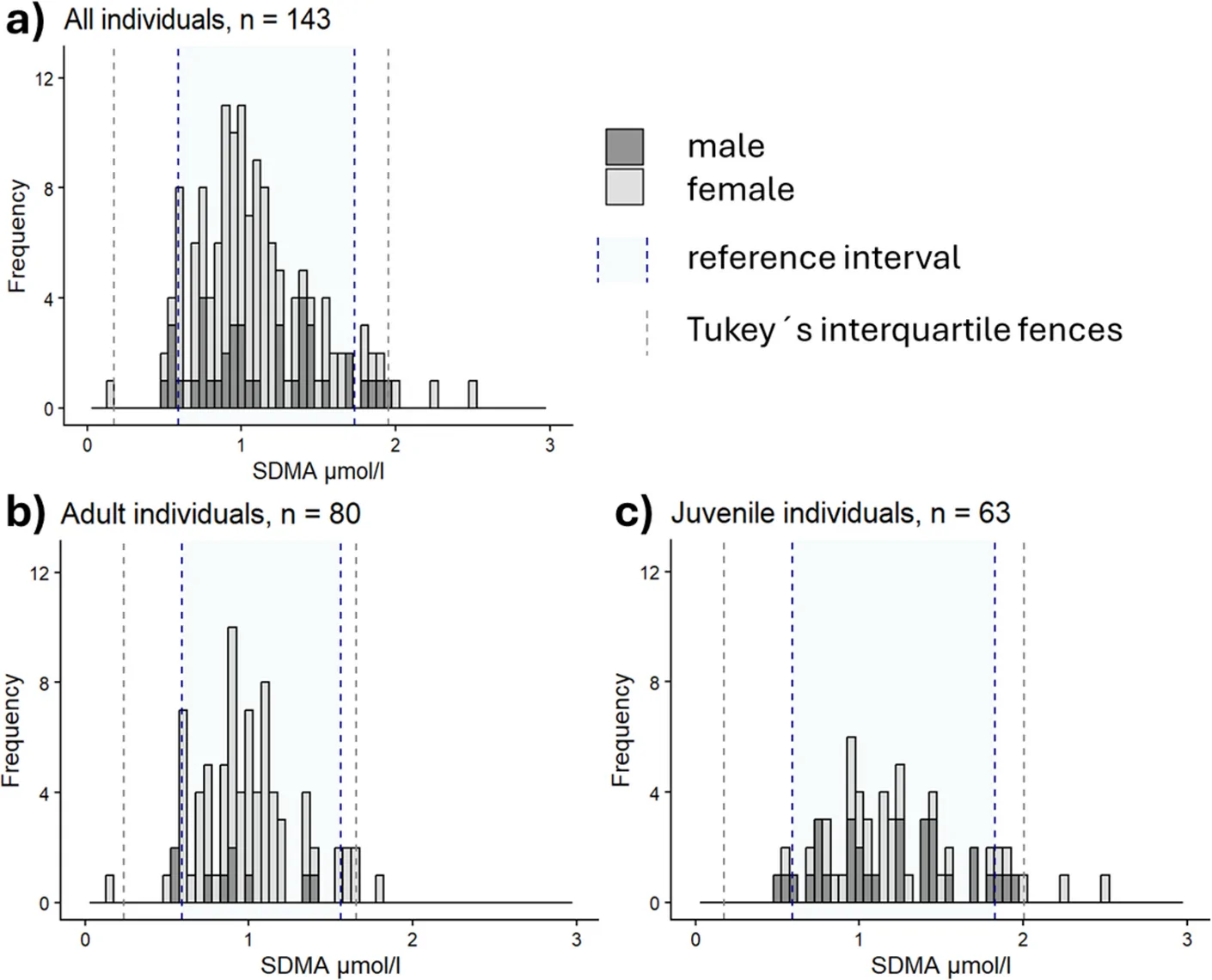
Calculation of reference limits for serum symmetric dimethylarginine (SDMA) concentrations in a population of 143 clinically healthy Lacaune sheep. Outliers were identified using Tukey’s interquartile fences and excluded where applicable. Reference limits were defined as the 5th and 95th percentiles of the non-outlier SDMA values. Calculations were performed for the entire population (**a**; 0.59–1.72 µmol/l), adults (**b**; 0.59–1.56 µmol/l), and juveniles under one year of age (**c**; 0.59–1.83 µmol/l)

Individuals were categorized by age into juvenile (< 1 year; 31 males, 32 females) and adult (≥ 1 year). The mean age of female sheep over 1 year was 4.7 years (σ = 1.98 years, *n* = 71), the mean age of male sheep over 1 year was 5.0 years (σ = 1.49 years, *n* = 9; Table 2). No sex-related differences in serum SDMA concentrations (*p* = 0.53, Fig. 1a) were observed during analysis of the complete dataset comprising all clinically healthy individuals (*n* = 143). When explored separately by age group, similarly, no statistically significant sex-related differences were observed in either adults or juveniles. However, juvenile animals exhibited significantly higher serum concentrations than adult sheep (*p* < 0.001, Fig. 1b). Prior to calculation of the reference interval, five outliers were identified using Tukey’s interquartile fences and excluded from the reference analysis. Based on the remaining 138 sheep, a reference interval of 0.59 (90% CI: 0.55—0.61) – 1.72 (90% CI: 1.64—1.82) µmol/l (mean = 1.09 µmol/l; median = 1.04 µmol/l; Fig. 2a) was established for serum SDMA in sheep. The measured values for this data set were distributed slightly right skewed. As significant differences between juvenile and adult animals were detected, reference calculations were additionally performed separately for adults (≥ 1 year) and juveniles (< 1 year). For adult animals, two of 80 individuals were identified as outliers. Based on the remaining 78 adult sheep, a reference interval of 0.59 (90% CI: 0.46—0.60) – 1.56 (90% CI: 1.39—1.65) µmol/l (mean = 0.99 µmol/l; median = 0.97 µmol/l) was established (Fig. 2b). By considering only juvenile animals, three of 63 individuals were identified as outliers. Based on the remaining 60 juvenile sheep, a reference interval of 0.59 (90% CI: 0.51—0.74) – 1.83 (90% CI: 1.83—2.24) µmol/l (mean = 1.18 µmol/l; median = 1.15 µmol/l) was determined (Fig. 2c). Although a normal distribution was assumed for both data sets by age group, reference interval calculation was nonparametric.

**Table 2:** Demographic data of the examined reference population and the clinical cohort

	Reference population	Clinical cohort
Breed	Lacaune	Several different breeds
Adult females	71	29
Juvenile females (< 1 year)	32	4
Adult males	9	9
Juvenile males (< 1 year)	31	9
Total number of individuals	143	51

### Clinical cohort

**Fig. 3:**
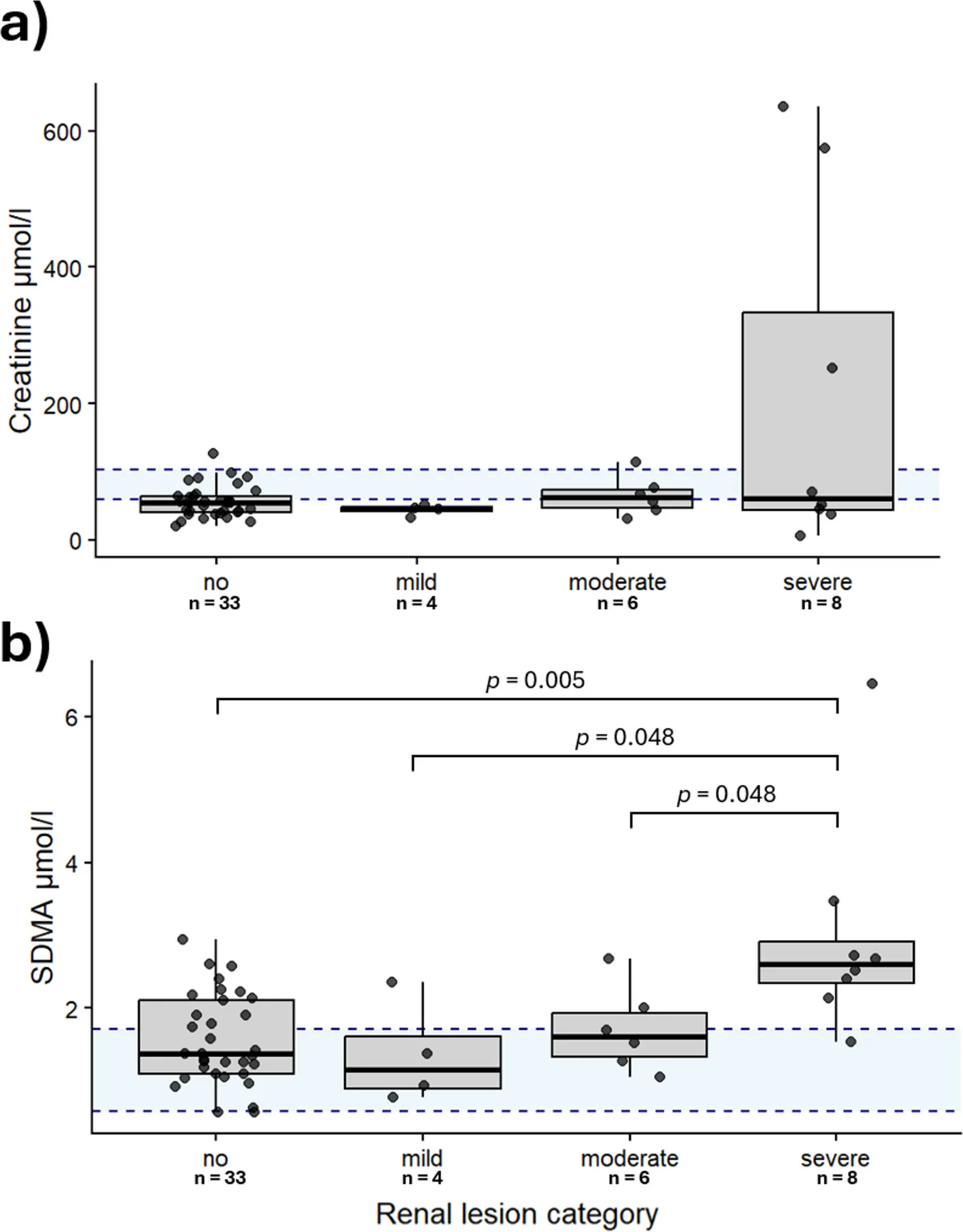
Comparison of serum creatinine and symmetric dimethylarginine (SDMA) concentrations in a population of 51 sheep of various breeds presented to a veterinary clinic and subjected to post-mortem histopathological examination. Individuals were categorised according to the severity of renal lesions. Reference intervals for creatinine [50] and SDMA (determined in the present study) are indicated as blue shaded regions in the background. No significant differences in creatinine concentrations were observed between groups using paired Wilcoxon tests. In contrast, SDMA concentrations were significantly higher in the group with severe renal lesions compared with the other categories

**Fig. 4:**
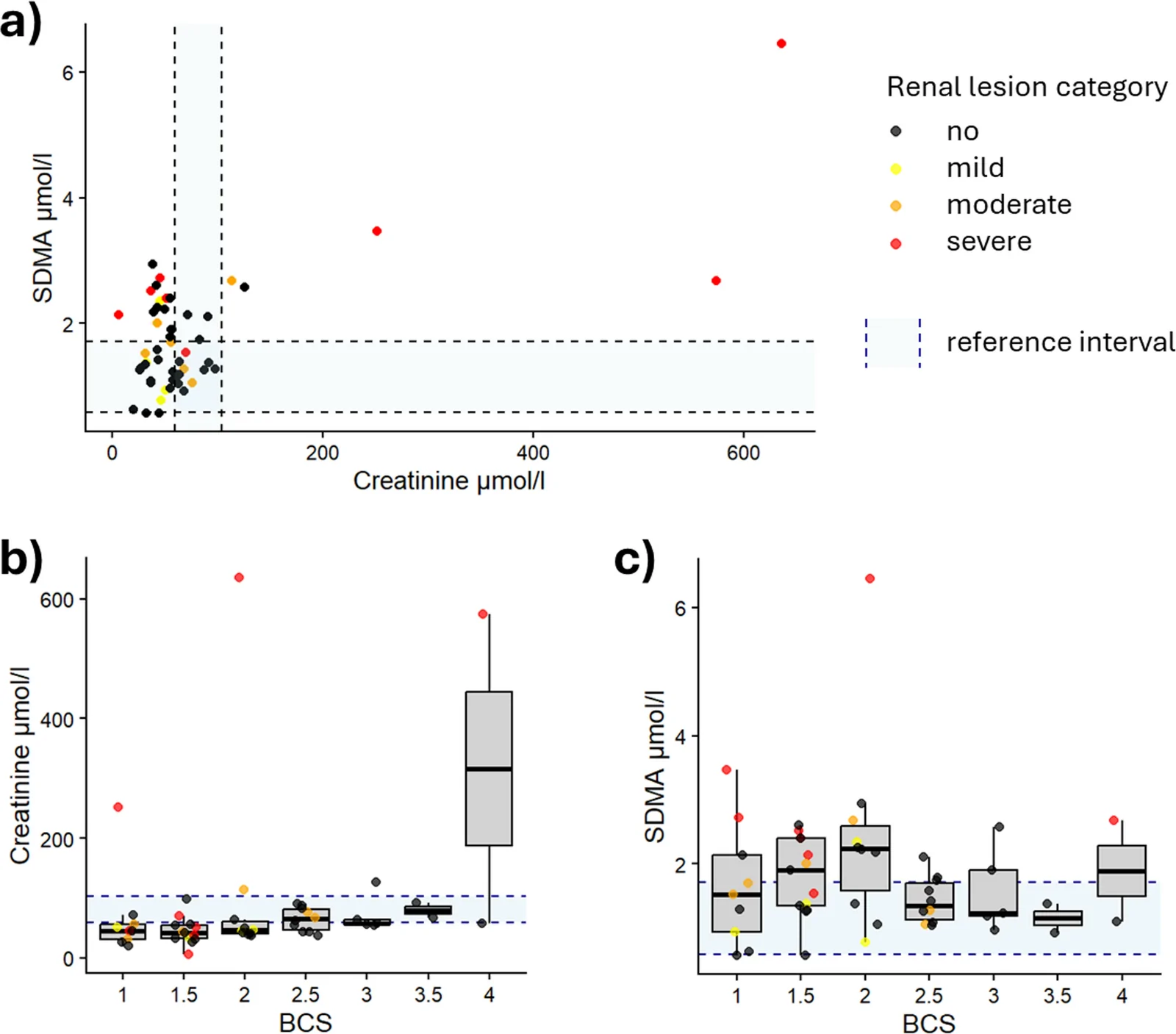
Direct comparison of serum symmetric dimethylarginine (SDMA) and creatinine concentrations in a population of 51 sheep of various breeds presented to a veterinary clinic and subjected to post-mortem histopathological examination. At lower creatinine concentrations, SDMA provides superior diagnostic resolution (**a**), likely due to the dependence of serum creatinine on muscle mass, approximated here by the clinically assessed body condition score (BCS; [49], **b**). This effect is markedly less pronounced for SDMA, where almost all individuals with severe renal lesions are identified by values above the reference interval, even at low BCS (**c**)

Serum creatinine concentrations did not differ significantly between animals of different renal lesion categories (paired Wilcoxon tests; *p* > 0.57; Fig. 3a). Using the reference interval reported by Tschuor et al. [50], none of the animals with mild lesions (0/4), one with moderate lesions (1/6), and three with severe lesions (3/8) exceeded the upper reference limit of 104 µmol/l. In contrast, serum SDMA concentrations were significantly higher in animals with severe renal lesions compared with those without histopathological abnormalities (*p* = 0.005) and with mild and moderate lesions (both comparisons: *p* = 0.048; Fig. 3b). Based on the reference interval established in this study, 1/4 animals with mild lesions, 3/6 with moderate lesions, and 7/8 animals with severe lesions exceeded the upper reference limit of 1.72 µmol/l. Of the 33 animals without renal lesions, 13 showed SDMA concentrations above 1.72 µmol/l. Among these 13 animals, 11 were euthanized and 2 died spontaneously. Final diagnoses included severe pneumonia (4/13), gastrointestinal infections (3/13), severe anaemia (2/13), leukaemia (2/13), uterine rupture (1/13), and listeriosis (1/13). By plotting serum creatinine and serum SDMA against body condition, creatinine exhibited lower levels observed in animals with poor body condition, whereas SDMA was not affected (Fig. 4b and c).

## Discussion

As reference intervals are a fundamental component of laboratory testing and form the basis for clinical decision-making, a standardized approach to their establishment is essential to ensure the reliability and interpretability of the results. The American Society for Veterinary Clinical Pathology (ASVCP) recommends that, regardless of data distribution, approximately 120 individuals should be included in a reference population that is demographically representative of the target population [18]. This requirement was met in the present study, with a total of 143 animals included. The distribution of sex and age, comprising a large proportion of young animals of both sexes, many adult females, and comparatively few adult males, reflects the demographic structure typically observed in commercial European sheep herds. Reference intervals were additionally calculated separately for age groups. Within these subgroups (80 adults and 63 juveniles) the recommended sample size could not be achieved. As the data within the age-specific groups approximated a normal distribution, the use of 90% confidence intervals for the estimation of reference limits can be considered acceptable according to ASVCP guidelines [18]. The central 90% reference interval defined by the 5th and 95th percentiles is, however, narrower than the conventional central 95% interval (2.5th–97.5th percentiles). Consequently, more values from a healthy reference population are expected to fall outside these limits, which should be considered when interpreting SDMA concentrations, particularly those close to the reference limits. As the reference population consists exclusively of animals of the dairy sheep breed Lacaune and originates from a single farm, the dataset is, however, limited regarding breed and origin. Caution is therefore advised when extrapolating the determined reference intervals to other populations, particularly meat breeds, which can be expected to have different metabolic patterns and variations in feeding and husbandry practices. Another factor to consider is that the inclusion criteria, based on clinical examination, body condition assessment, and serum creatinine concentration, may inadvertently allow the inclusion of unhealthy individuals.

The measurement of SDMA in serum and other biological substrates can be performed using different analytical methods. Considered as gold standard is the measurement by liquid chromatography–tandem mass spectrometry (LC–MS/MS; [29]). However, measurement by ELISA is far more commonly used in routine diagnostics, as commercial kits from several manufacturers (e.g. IDEXX Laboratories, Westbrook, ME, USA and DLD Diagnostika GmbH, Hamburg, Germany) are available and costs per sample are substantially lower [16]. It should be noted, however, that ELISA-based measurements may yield slightly higher SDMA concentrations compared with LC–MS/MS [32]. Additionally, the ELISA used in this study (DLD) has thus far only been validated for dogs and cats. Consequently, the establishment of method-specific reference intervals is particularly important and validation of the method against the gold standard is strongly recommended [14, 27].

The reference interval determined in the present study using an ELISA (DLD) assay, ranging from 0.59 to 1.72 µmol/l for SDMA in the serum of clinically healthy Lacaune sheep of both sexes and different ages, is higher than most reference intervals previously reported for ELISA-based measurements in other domestic species. Lower reference ranges have been described in carnivores such as dogs (0.42–0.74 µmol/l; [32]), cats (0.00–0.74 µmol/l,Idexx [25]), and ferrets (0.15–0.40 µmol/l [36]), as well as in herbivores including horses (0.42–0.74 µmol/l [46]) and rabbits (0.21–0.95 µmol/l [48]). As the assay has not yet been validated for sheep against LC–MS/MS, this observation might be method-associated. For domestic ruminants, little information is available on physiological SDMA concentrations to date. For cattle, some data have been generated within the context of clinical studies. Validated reference intervals, however, have not yet been established. Bronzo et al., [11], for example, compared SDMA concentrations between healthy cows and animals affected by mastitis and reported mean values of approximately 0.11 µmol/l. In another study, serum SDMA concentrations measured by quantitative immunoassay (IDEXX Catalyst Analyzer, IDEXX laboratories, Memphis, Tennessee, USA) were compared between healthy Brown Swiss and Holstein cows. Brown Swiss animals showed significantly higher SDMA concentrations (≈ 0.83 µmol/l) than Holsteins (≈ 0.66 µmol/l), without any indication of impaired renal function [28]. For sheep, no published data on SDMA concentrations are currently available. In goats, the domestic species most closely related to sheep, a reference interval of 0.4–1.3 µmol/l was recently reported by Camacho et al., [12], measured by a quantitative immunoassay (IDEXX) based on 55 adult animals of both sexes and different breeds. In addition, a metabolomics study in 24 adult Saanen goats reported serum SDMA concentrations measured by LC–MS/MS ranging from 0.53 to 1.48 µmol/l [33]. Although these values were determined with a different methodology and are somewhat lower than the reference limits established in the present study for Lacaune sheep across all age groups, they are broadly comparable with the reference interval determined here for adult animals (0.59–1.56 µmol/l). The generally higher SDMA concentrations observed in small ruminants, as suggested by the currently limited body of data, may potentially be associated with the comparatively large gastrointestinal tract of these species. In relation to body weight, the digestive tract with its high cellular mass may produce disproportionately large amounts of nuclear metabolism by-products, such as SDMA.

Consistent with the findings of the present study, no significant differences in serum SDMA concentrations between sexes have been reported in studies of other animal species [40]. It should be emphasised, however, that the male portion of the population studied here consisted overwhelmingly of juvenile males (31/40). A sex-specific difference is, however, expected to be more evident in adult animals. Significant differences between age groups were, however, observed. Serum SDMA concentrations in juvenile sheep (0.59–1.83 µmol/l) were significantly higher than those measured in adult animals (0.59–1.56 µmol/l). Similar age-related effects have previously been reported in horses, dogs, and cats, in which juvenile individuals exhibited significantly higher serum SDMA concentrations than adults [10], IDEXX [25], Siwińska et al., [46]). The underlying mechanisms remain incompletely understood. However, it has been proposed that age-related differences in arginine methylation and protein metabolism may contribute to increased SDMA release in young animals, whereas renal excretion already appears to be functionally comparable to that of adults [3].

For several reasons, the evaluation of SDMA in comparison with creatinine in this study, based on a retrospective analysis of 51 clinically diseased and pathologically examined animals, must be considered only indicative and cannot replace the validation of the parameter for sheep using a larger study population and a gold-standard method such as measurement of glomerular filtration rate. Due to demographic differences (e.g., breed composition, sex distribution, and age distribution) alone, the clinical cohort is only partially comparable to the reference population. Furthermore, the underlying histopathological findings do not always reflect renal function, as prerenal effects are rarely captured. This may explain why some animals without renal lesions exhibited elevated serum SDMA concentrations. Such findings are not unexpected in this population, which consisted exclusively of animals that died or were euthanized within 48 h of sample collection. Perfusion disturbances due to dehydration or shock in severely ill individuals are not uncommon.

The dataset, however, provides strong evidence that SDMA outperforms creatinine in animals with poor body condition. Individuals with severe renal lesions and low serum creatinine concentrations showed serum SDMA levels above the established reference interval (Fig. 4a). This is likely due to the dependence of serum creatinine on muscle mass, approximated here by the clinically assessed body condition score (BCS; [49], Fig. 4b). This effect is considerably less pronounced for SDMA, as nearly all individuals with severe renal lesions are identified by concentrations above the reference interval, even at low BCS (Fig. 4c).

## Conclusions

Compared with reference intervals reported for most other domestic species, the measured serum SDMA concentrations in Lacaune sheep are slightly higher. The established reference range for adult sheep is, however, comparable with previously published data for goats. As SDMA is a metabolite of all nucleated cells, the assessment of the renal function via this parameter should also for sheep be more sensitive than via creatinine or blood urea nitrogen (BUN). The evaluation of the clinical cohort, however, provides only preliminary guidance and represents a first step toward the validation of SDMA for diagnostic use in this species. Further studies are clearly necessary. Nonetheless, the application of the serum SDMA reference interval established here suggests good diagnostic utility, particularly in animals with poor body condition. Separate reference intervals should be considered for juvenile and adult animals, as these differ significantly. An important limitation restricting the use of this biomarker in sheep is economic: at present, SDMA measurement by ELISA, although less costly than LC–MS/MS, remains considerably more expensive than the inexpensive photometric assessment of creatinine.

## Supplementary Information

Supplementary Material 1. Supplementary File 1: Original data of 51 deceased sheep patients presented to the veterinary clinic for small ruminants between 2018 and 2022. The dataset includes demographic attributes (sex, age), clinical data (Body Condition Score [BCS], final clinical diagnosis), laboratory parameters (serum creatinine concentration in µmol/l, serum symmetric dimethylarginine [SDMA] in µmol/l) and histopathological findings.

## Data Availability

Data is available upon request.
